# Supplementary figures and images for: Nuclear speckles regulate HIF-2α programs and correlate with patient survival in kidney cancer
Source: bioRxiv. 2023 Sep 16:2023.09.14.557228. Preprint. [Version 1] doi: 10.1101/2023.09.14.557228 (PMC10515914; doi:10.1101/2023.09.14.557228)

# Figure S1

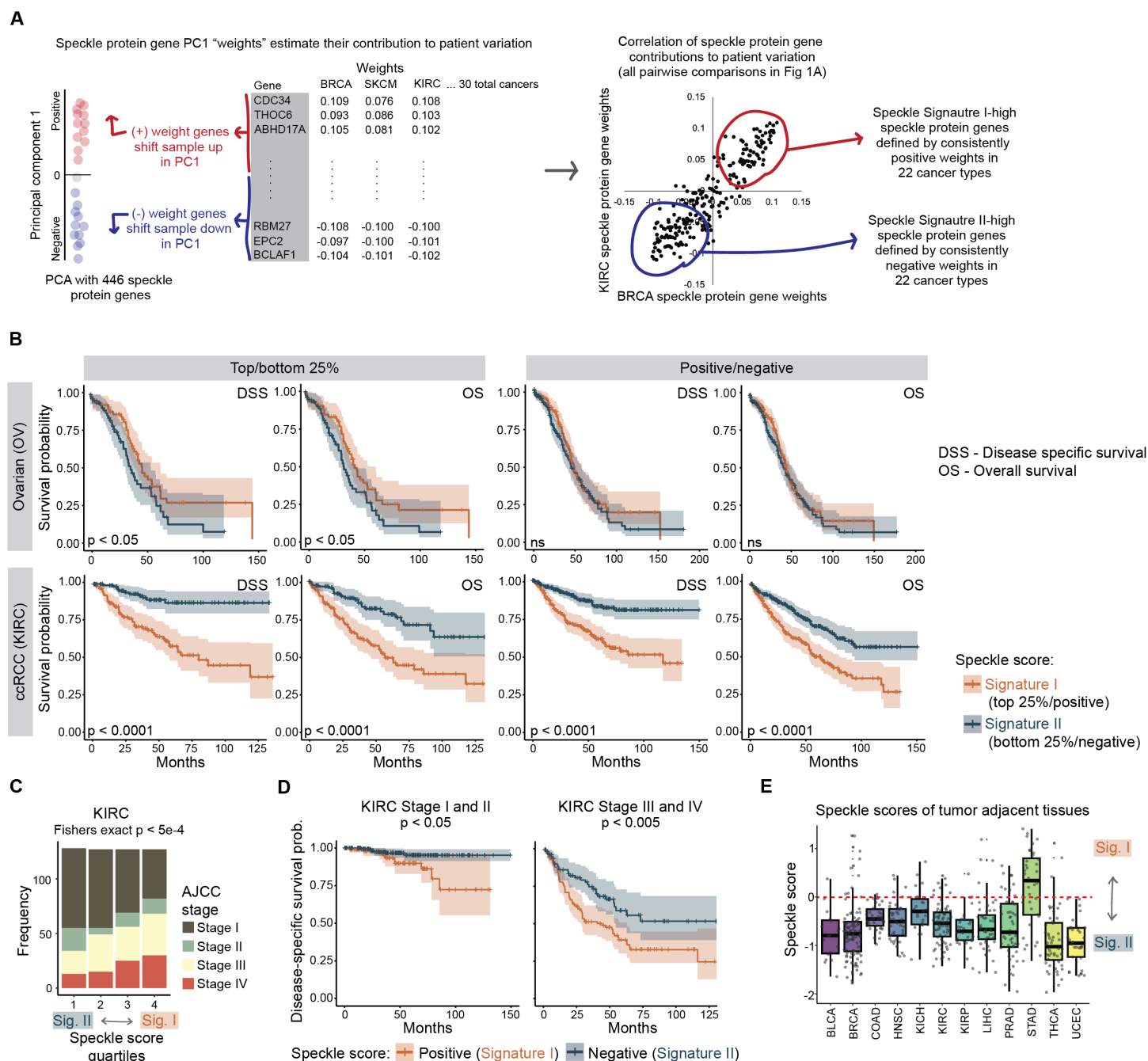



## Figure S3

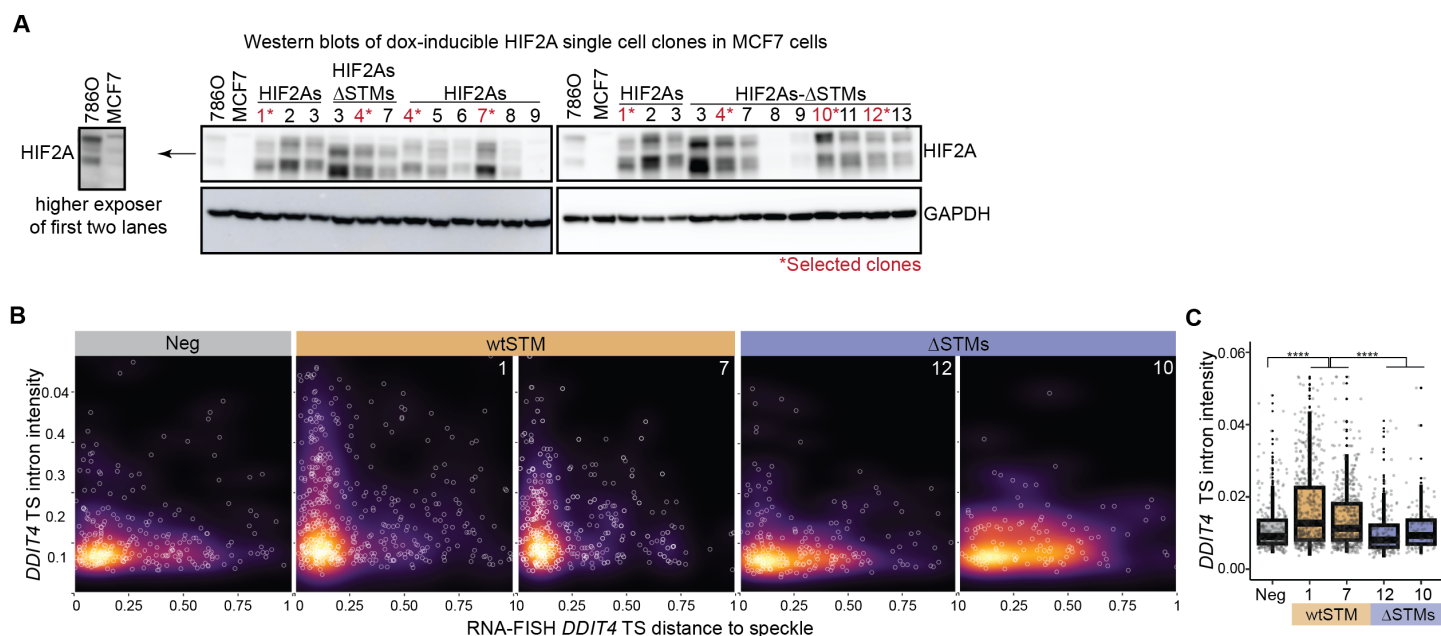

## Figure S4

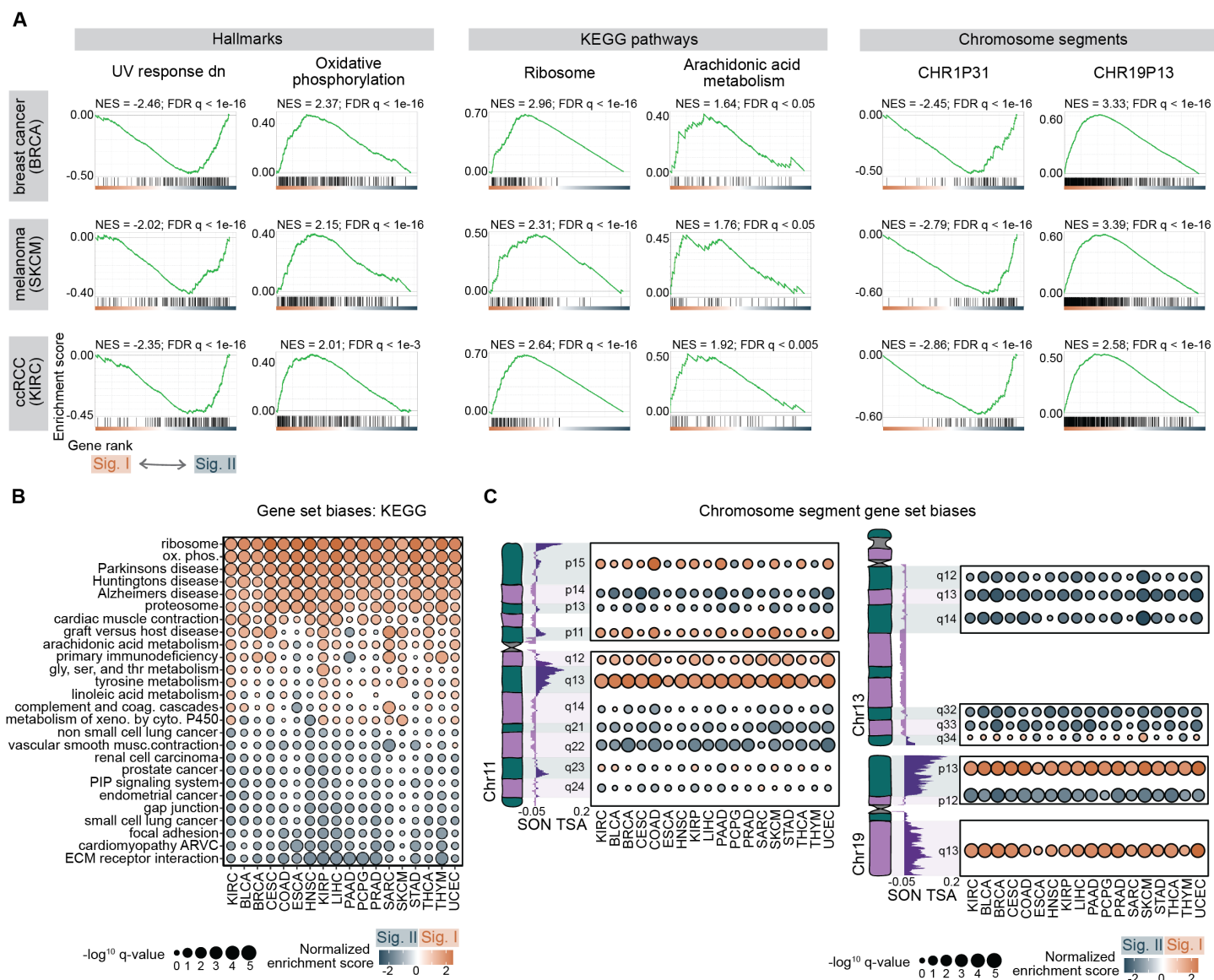

## Figure S5

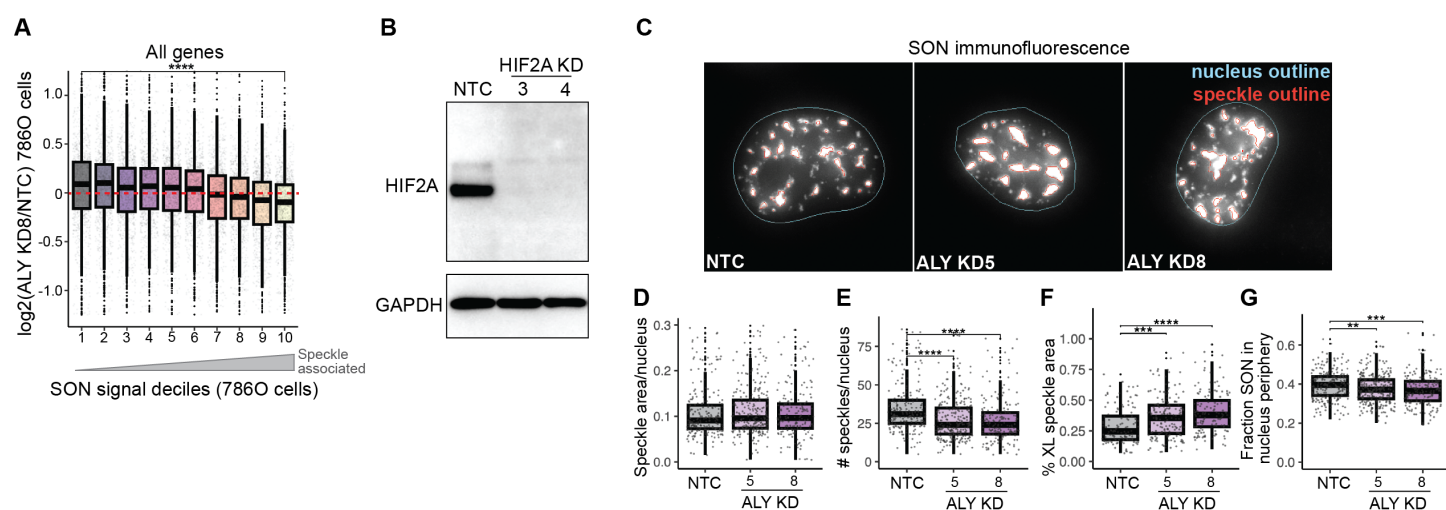

Supplement: Supplement 4 — Figure S1. Supplement to Figure 1. A) Schematic showing how speckle protein genes with high contributions to patient variation were selected based on Principal Component 1 (PC1) weights. B) Kaplan Meier disease-specific (DSS) or overall survival (OS) analysis for ovarian cancer (OV, top) and ccRCC (KIRC, bottom) splitting individuals based on the top/bottom quartiles of speckle score (left) or on positive versus negative speckle score (right). C) Tumor AJCC stage frequency of ccRCC (TCGA KIRC cohort) tumors based on speckle scores split into quartiles. First quartile are the most Signature II tumors; fourth quartile are the most Signature I tumors. D) Kaplan Meier plots for early (left) and late (right) stage ccRCC (TCGA KIRC cohort). Patients were split into Signature I (positive speckle score) and Signature II (negative speckle score). E) Speckle scores of tumor adjacent normal tissues. Speckle scores are calculated independently for each tissue/cancer type. See Table S1 for survival statistics for each cancer and Github (https://github.com/katealexander/speckleSignature.git) for detailed instructions, scripts used, and additional files generated relating to speckle signature calculations and survival analysis. Figure S2. Supplement to Figure 2. A) EMBOSS Matcher alignment showing the best match local motif between p53 (amino acids 62-90) and HIF-2α (amino acids 450-478). B) Clustal Omega alignment of HIF-1α with HIF-2α with MNView visualization. HIF-2α STMs are boxed in red. C) Zoomed in view of HIF-2α STMs in HIF-1α HIF-2α alignment from B. D) Criteria for de novo identification of speckle targeting motifs (STMs). Detailed instructions can be found on Github (https://github.com/katealexander/speckleTargetingMotif.git). E) Scatterplot showing relationship between SON TSA-seq and SON Cut&Run normalized counts, averaged over two replicates. RP – Pearson’s R. F) Venn diagram showing the base-pair overlap between regions called as decreasing SON signal in SON TSA-seq [file NIHPP2023.09.14.557228v1-supplement-4.pdf]
